# Implications and Current Limitations of Oogenesis from Female Germline or Oogonial Stem Cells in Adult Mammalian Ovaries

**DOI:** 10.3390/cells8020093

**Published:** 2019-01-28

**Authors:** Jessica J. Martin, Dori C. Woods, Jonathan L. Tilly

**Affiliations:** Laboratory of Aging and Infertility Research, Department of Biology, Northeastern University, Boston, MA 02115, USA; martin.je@husky.neu.edu (J.J.M.); d.woods@northeastern.edu (D.C.W.)

**Keywords:** oogenesis, oocyte, germ cell, germline stem cell, oogonial stem cell, meiosis, ovary, fertility

## Abstract

A now large body of evidence supports the existence of mitotically active germ cells in postnatal ovaries of diverse mammalian species, including humans. This opens the possibility that adult stem cells naturally committed to a germline fate could be leveraged for the production of female gametes outside of the body. The functional properties of these cells, referred to as female germline or oogonial stem cells (OSCs), in ovaries of women have recently been tested in various ways, including a very recent investigation of the differentiation capacity of human OSCs at a single cell level. The exciting insights gained from these experiments, coupled with other data derived from intraovarian transplantation and genetic tracing analyses in animal models that have established the capacity of OSCs to generate healthy eggs, embryos and offspring, should drive constructive discussions in this relatively new field to further exploring the value of these cells to the study, and potential management, of human female fertility. Here, we provide a brief history of the discovery and characterization of OSCs in mammals, as well as of the in-vivo significance of postnatal oogenesis to adult ovarian function. We then highlight several key observations made recently on the biology of OSCs, and integrate this information into a broader discussion of the potential value and limitations of these adult stem cells to achieving a greater understanding of human female gametogenesis in vivo and in vitro.

## 1. Introduction: A Brief History of Postnatal Oogenesis in Mammals

Before the 1950s, the question of whether mammalian females could generate new oocytes during adulthood had been constructively debated for years, with evidence in favor of and against the concept of postnatal oogenesis mired in some degree of confusion. For example, in 1945 Everett noted that “*morphological studies relating to the origin and differentiation of the definitive germ cells in vertebrates have … resulted in conflicting views. In many instances two or more competent investigators who have studied the same form have reached different conclusions*” [1]. One of the earliest reports on this subject was published in the late 1800s by Wilhelm von Waldeyer-Hartz, a German anatomist who hypothesized based on histological surveys that no additional oocytes were produced in the ovaries after the perinatal period [2]. Although subsequent studies in the early 1900s generated evidence that countered this belief [3,4,5,6,7,8,9,10], the prevailing view in the field remained mostly in alignment with Waldeyer’s earlier opinion. After more than 5 decades of scientific inquiry and discussion, this debate came to an abrupt halt in 1951, when Zuckerman concluded from his review of the scientific literature at the time that oocyte formation does, in fact, irreversibly cease by the time of birth [11].

While never deviating from this opinion [12], Zuckerman later clarified that in stating this conclusion he meant “*no more than none of our experimental or observational findings* [at that time] *is inconsistent with the hypothesis first stated by Waldeyer*” [13]. However, Zuckerman may have approached the controversy with a pre-conceived opinion of how the debate should be settled. Specifically, in discussing earlier work by Allen [5], Zuckerman stated that this study held the “*distinction to be the first to make a serious dent in what Pearl and Schoppe* [14] *correctly described as the ‘basic biological doctrine that during the life of the individual there neither is nor can be any increase in the number of primary oocytes beyond those originally laid down when the ovary was formed*” (bold typeface added by the authors for emphasis, reference citation added by the authors) [11]. A few studies continued to sporadically surface over the next 10 years that reported experimental findings discordant with Zuckerman’s conclusion [15,16,17], but history shows that the opinion of Zuckerman rapidly became cemented in the field as fact. In turn, studies contrary to this belief were, if one carefully evaluates the literature at the time, largely ignored. Unfortunately, a fundamental question was apparently never posed. Why was a ‘dogma’ so critically important to the field of reproductive biology based solely on Zuckerman’s opinion that none of the data he considered at the time were inconsistent with Waldeyer’s initial hypothesis [2], rather than on direct experimental findings proving that adult mammalian females are incapable of oogenesis? Using Zuckerman’s reasoning [11], the dogma of a fixed pool of oocytes being set forth at birth would be invalidated simply by scientific evidence inconsistent with the idea that this population of germ cells is not subject to renewal in postnatal life. As discussed above, such evidence clearly existed [3,4,5,6,7,8,9,10,15,16,17], but was still discounted.

## 2. Germline Stem Cells in Adult Female Flies—So, Why Not Female Mammals?

The occurrence of oogenesis in ovaries of adult fruit flies (*Drosophila melanogaster*) was described in the mid-1950s [18], which followed on the heels of Zuckerman’s opinion piece [11] but was nonetheless widely accepted, probably because the work employed an insect model system. The subsequent identification of female germline stem cells (GSCs) as the source of new oocytes in adult fly ovaries ignited considerable interest in the model for detailed genetic studies of developmental and stem cell biology [19,20,21]; however, the relevance of this work to mammalian female reproduction was generally considered minimal at best. By comparison, a strong and consistent parallel was drawn between the role of GSCs in supporting spermatogenesis in the testes of adult males throughout evolution, without any questions raised about the central importance of stem cells to male gametogenesis from flies to humans [22,23,24,25,26]. To make the case that, during evolution, males of only mammalian species would move towards endowment of a set population of spermatozoa in their testes at birth, rather than continue to produce a new complement of sperm every day as their ‘less-evolved’ counterparts have done for thousands of years, would have most likely been ridiculed. However, even with the knowledge that active oogenesis occurs in adult ovaries of not just insects [27,28,29] but of vertebrates as well [14,30,31,32,33,34,35], many scientists in the field of reproduction remained entrenched in the belief that female mammals somehow diverged from ‘lower’ species to become incapable of generating new oocytes after birth. In hindsight, the premise that evolutionary pressure drove deviation of female gamete production in mammals away from a renewable resource model, which serves to maximize the chances of species survival due to the constant availability of ‘fresh’ gametes for reproduction, to one characterized by endowment of a fixed population of gametes at birth makes very little sense [36].

In 2004, a study was published using mice as a model system, which offered several different lines of experimental findings inconsistent with the opinion of Zuckerman [11] that mammalian females are incapable of postnatal oogenesis and folliculogenesis [37]. Not surprisingly, this report ignited a new round of debate over the concept of postnatal oogenesis in mammals. Evidence in support of the idea that adult female mice contain mitotically-active, oocyte-forming germ cells—which were later termed oogonial stem cells (OSCs) because of their functional similarity to spermatogonial stem cells (SSCs) in males, was cautiously viewed by some as a major advancement in the field of reproductive sciences [38], while others outright dismissed the work ([39]; see also [40]). The intensity of the debate was raised further by a second study on neo-oogenesis in mice published in 2005 [41], prompting a new round of opinion pieces on the matter [42,43] and follow-up investigations with comparable results but conflicting conclusions [44,45,46,47]. Additional experimental studies reporting observations both for [48,49] and against ([50]; see also [51]) the possibility of postnatal oogenesis in mammals were published over the next few years, along with several new opinion pieces as each side of the debate took stronger positions on the validity of their views [52]. The tone of the debate changed dramatically, however, in 2009 when primitive germ cells, capable of both ex vivo expansion (mitotically competent) and in vivo differentiation into functional eggs (meiotically competent) that give rise to viable offspring following intraovarian transplantation into recipient female mice, were isolated from postnatal mouse ovaries [53]. These findings, viewed by many as a major stepping stone in the history of OSC research, prompted some skeptics of the earlier work to reconsider their views [54,55,56]; however, others continued to voice serious doubts, if not complete disbelief, over the validity of these studies ([57,58,59]; see also [60]).

**Figure 1:**
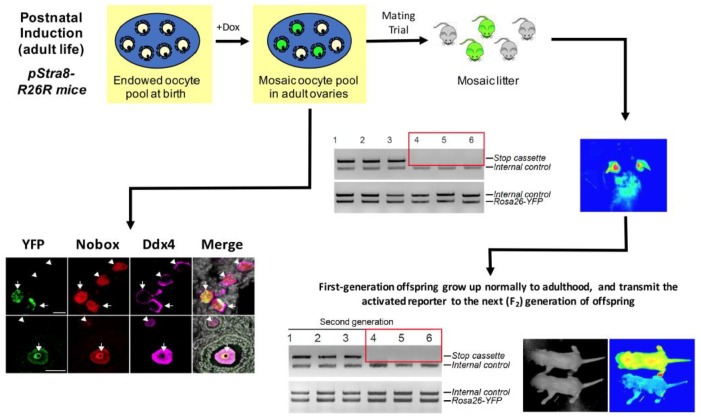
Oocytes formed during adult life in mice contribute directly to offspring production. Schematic representation of an inducible genetic lineage-tracing model to ‘mark’ new oocytes formed during the doxycycline (dox) induction phase, which specifically activates a triple-transgenic Cre-*loxP*–based reporter system tied to *stimulated by retinoic acid gene 8* (*Stra8*) expression in pre-meiotic germ cells (viz. OSCs) committing to meiosis followed by de novo oogenesis. Portions of this figure were adapted with permission from Wang et al. [84].

Nonetheless, against a backdrop of continued debate and scattered reports claiming from negative data to refute the existence of OSCs and the ability of adult mouse ovaries to generate new oocytes ([61,62,63,64,65]; see also [66,67,68,69]), studies of OSCs in rodent models continued to populate the scientific literature, with nearly 30 published primary studies now available reporting on the characteristic features and functional properties of these cells in the context of postnatal oogenesis in mice and rats [37,41,49,53,66,67,68,70,71,72,73,74,75,76,77,78,79,80,81,82,83,84,85,86,87,88,89,90,91,92]. These efforts have recently been paralleled by similar studies of OSCs in adult ovaries of cows [93,94], non-human primates [64,69] and pigs [95,96], underscoring the evolutionary conservation of the findings across diverse mammalian species. Importantly, the use of intragonadal transplantation-based approaches to establish the functional capacity of rodent OSCs to generate eggs that fertilize to produce embryos and offspring [53,67,72,73,78,80,84,85] was recently extended by inducible suicide gene-based targeted ablation and inducible genetic fate-mapping studies in mice [81,84]. Among other things, these lines of investigation demonstrated that new oocytes are formed in the ovaries during adult life [81,84], and that some of these newly-formed oocytes contribute directly to the pool of eggs used for natural reproduction [84]. It is worth mentioning that these types of genetic approaches to document the physiological relevance of postnatal gametogenesis in female mammals are still lacking in adult males. This seems counter to the beliefs of some scientists that the published data are not rigorous enough to firmly establish the significance of de novo oogenesis to adult ovarian function and natural female fertility under normal physiological conditions (Figure 1).

## 3. Discovery and Characterization of Human OSCs

Arguably, aside from the 2004 study of Johnson et al. that initially prompted a re-thinking of Zuckerman’s five-decade-old dogma [37], along with the subsequent demonstration that purified OSCs can generate functional eggs in vivo [53], the most significant advancement in this field came with the successful purification of a rare population of cells from ovarian cortical tissue of reproductive-age women that possessed the same fundamental germline characteristics and oocyte-forming features ascribed to the cells identified as OSCs in rodents [73]. The outcomes of this study, which have since been independently verified by studies of human OSCs from three other laboratories ([97,98,99]; see also [93,100,101]) and extended by early clinical studies of human OSCs with women undergoing assisted reproduction ([102,103]; reviewed in [104]), opened the novel prospect of applying the principles of stem cell-based regenerative medicine to the management of human female infertility and ovarian failure [105,106,107,108]. Although detailed discussions of the biology and potential clinical uses of human OSCs are beyond the scope of this article, we will briefly highlight several key observations made from work with human OSCs over the past several years that underscore the central features of these cells.

We will begin with the method employed to obtain OSCs from ovarian cortical tissue of both pre- and post-menopausal women [73,97,98,99,101]. The underlying approach relies on the use of an antibody against the C-terminus of the germ cell protein, DEAD-box polypeptide 4 (DDX4) [109,110,111], to identify and purify those cells with externalized (extracellular) exposure of this specific domain of the protein by fluorescence-activated cell sorting (FACS) or magnetic-assisted cell sorting (MACS) [53,68,73,112]. The cells obtained from human ovarian tissue by this method exhibit many characteristic germline features that closely align with OSCs isolated from adult mouse ovaries. Furthermore, the DDX4 antibody-based sorting approach has been successfully utilized by four different groups to obtain OSCs from adult human ovarian tissue, all of which have consistently reported the isolation of a rare population of viable germ cells, expressing an externalized epitope of DDX4, which could be expanded and studied in vitro [73,97,98,99,101]. We feel this is important to emphasize since the use of C-terminal-directed DDX4 antibodies to isolate OSCs has been questioned by some because of the widespread belief that DDX4 is a cytoplasmic protein in all germ cells ([64,65]; see also [69]).

Unfortunately, the basis of this belief is rooted in studies conducted many years before OSCs had been identified [109,110,111], and thus this specific type of primitive female germ cell was not yet known to exist, much less be available for inclusion in these early analyses. Coupled to this issue is potential confusion over what exactly ‘DDX4-positive’ means in the context of OSC isolation, since all germ cells are arguably DDX4-positive. We have proposed in prior studies that the terminology be modified to refer to OSCs isolated by MACS or FACS using this approach as extracellular DDX4 (ecDDX4)-positive [66]. This acronym (viz. ecDDX4-positive) would distinguish these cells from all other types of germ cells, including oocytes, in which DDX4 is expressed (DDX4-positive) but retained completely within the cytoplasm and, thus, the protein would not be identified in live (non-permeabilized) cell sorting platforms by C-terminal DDX4 antibodies [73]. Building on extensive validation work to establish the fidelity of this approach to purify OSCs [68,73,112], including computational modeling [53,68], immuno-microbead technologies [73], dual-antigen/single-protein FACS [73] and mass spectrometry [69], Clarkson et al. [98] recently verified once again the presence of externalized DDX4 on human OSCs using FACS coupled to a well-established stem cell marker, aldehyde dehydrogenase (ALDH1) activity. Moreover, the inclusion of ALDH1 activity as a secondary endpoint revealed the existence of subpopulations of ecDDX4-positive cells in adult human ovaries that differ based on relative levels of ALDH1 activity [98]. Although the significance of this is not yet entirely clear, it should encourage further work to determine if OSCs isolated based on ecDDX4 expression vary in their degree of ‘stemness’, like that reported for hematopoietic stem cell heterogeneity in bone marrow [113]. Irrespective, three groups have independently reported that human OSCs, when allowed to interact with ovarian somatic cells, are capable for forming immature oocytes that generate follicles in adult human ovarian tissue xenografts [73,97] or follicle-like structures in fetal human ovarian tissue re-aggregates [98].

**Figure 2:**
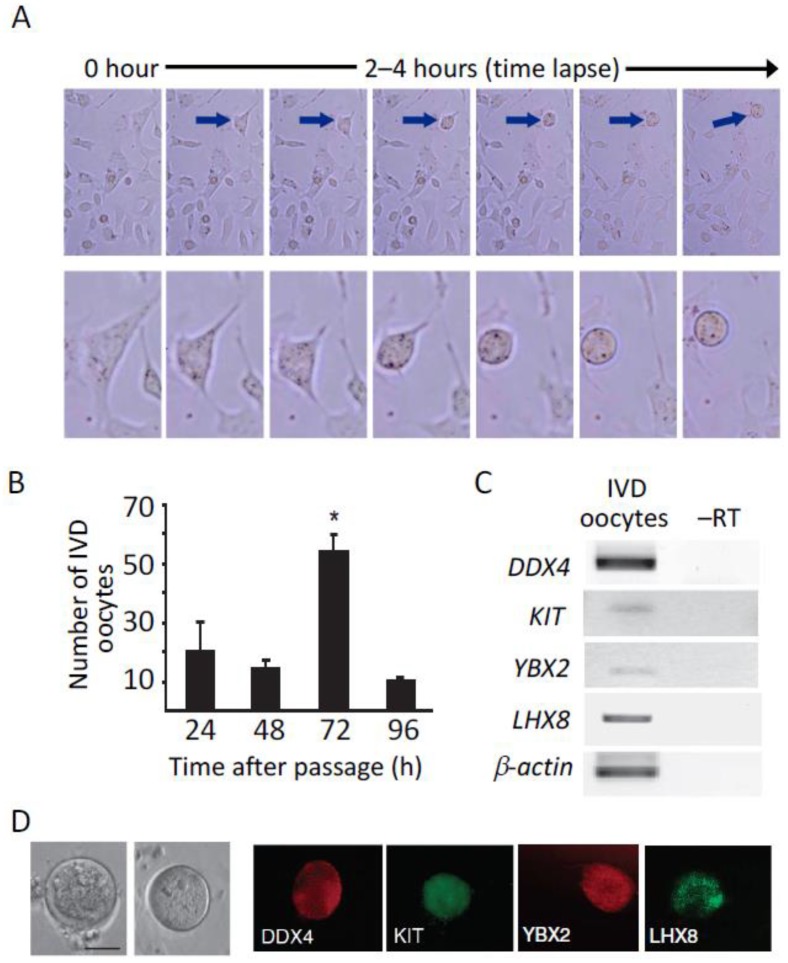
In vitro oogenesis from human OSCs. (**A**) Time lapse images of human OSCs in culture, with a typical OSC (blue arrow) followed as it undergoes progressive differentiation into an IVD oocyte (oocyte-like cell). (**B**) Numbers of IVD oocytes formed in human OSC cultures over time post-passage. (**C**) Expression analysis of germ cell (*DDX4*) and oocyte (*KIT* oncogene or *KIT*; *Y-box protein 2* or *YBX2*, also referred to as *MSY2* or *CONTRIN*; *LIM homeobox protein 8* or *LHX8*) marker genes, as well as of *β-actin* expression as a loading control, in IVD oocytes collected from human OSC cultures (–RT, PCR analysis performed on the RNA template without reverse transcription, as a control to rule out genomic DNA amplification). (**D**) Representative images of human IVD oocytes by light microscopy (two left panels; scale bar, 50-µm), and by immunofluorescence microscopy for the presence of DDX4, KIT, YBX2 and LHX8 proteins. Portions of this figure were adapted with permission from White et al. [73].

Another interesting property of human OSCs reported across studies is the capacity of these cells, when cultured in vitro as actively-dividing germ cells, to spontaneously differentiate into large ovoid cells that express markers of meiotic progression (SYCP3, synaptonemal complex protein 3) and of oocytes (GDF-9, growth differentiation factor-9). While this characteristic feature of cultured OSCs has previously been studied in detail [73] (Figure 2), and utilized as an informative bioanalytical endpoint for studies of adult female gametogenesis in mouse models [75,84] and in comparative studies of murine versus human OSCs [114], Silvestris et al. [99] recently took this observation one step further using fluorescence in situ hybridization (FISH) and single cell analysis to document meiotic progression in the oocyte-like cells, or in-vitro—derived (IVD) oocytes, formed by human OSCs in culture. An indisputable feature of germ cells progressing through meiosis is an initial replication of chromosomes in the diploid precursor cells during interphase, with identical sister chromatids held together by centromeres. At prophase-I, each chromosome has paired with its corresponding homologous chromosome to form a tetrad, with four chromatids contained in each tetrad. Immature oocytes remain arrested at this stage of meiosis-I until progression is signaled to resume in mature follicles following the luteinizing hormone surge in reproductive-age females. By the time the egg has been ovulated, it has completed meiosis-I and become arrested once again during metaphase of meiosis-II, having extruded the first polar body. The second polar body is then extruded at syngamy, leading to completion of meiosis-II in the fertilized egg and the subsequent formation of a diploid zygote following fusion of the male and female pronuclei [115].

It is widely believed that this entire process of female gamete maturation in vivo is choreographed by the follicular granulosa cells surrounding each oocyte. Without the influence of their appropriate somatic cell partners (viz. granulosa and granulosa-cumulus cells), meiotic progression in oocytes continues unabated, bypassing key arrest checkpoints [115]. This is important to highlight when evaluating the ability of OSCs to produce IVD oocytes in culture, since the cells are maintained in the absence of granulosa cells and, thus, are not subject to the meiotic ‘brakes’ normally applied to in-vivo—maturing oocytes [68,73,84,89,97,99]. By tracking chromosomal content through FACS analysis [116], White et al. [73] reported the first evidence that mouse and human OSCs, when cultured in vitro, generate a rare population of haploid (1*n*) cells. These data were supported by parallel findings of punctate localization of the meiosis-specific DNA recombinase, dosage suppressor of mck1 homolog (DMC1), and the meiotic recombination protein, SYCP3, in nuclei of cells in human OSC cultures, as well as extensive gene profiling-based characterization of IVD oocytes to confirm expression of a spectrum of classic oocyte markers [73] (Figure 2). Silvestris et al. [99] significantly extended these prior results by FISH-based assessment of chromosomes X and 5 in single cells isolated based on size differences from human OSCs maintained in vitro. As expected, two distinct signals were observed for each chromosome in the ‘small’ cells or proliferative OSCs, consistent with these cells having a diploid status; however, the ‘large’ oocyte-like cells exhibited a single signal for each chromosome, indicative of these cells having reached formal haploid status [99].

We feel these latter findings are important to highlight for two principal reasons, the first being verification that, using a universally-accepted technology for assessing chromosomal numbers in cells, human OSCs are indeed capable of completing meiosis to produce haploid female germ cells [99]. The second is related to the utility of human OSCs in culture to serve as a bioassay or screening platform for identification of factors that drive human oocyte formation [107,114]. Since this approach has already proven successful in rodent OSC models [75], with predictive value for in vivo oogenesis [84], this may be of great service to the design and optimization of technology platforms directed at the generation of human eggs from stem cells in vitro (see concluding section below for further discussions).

## 4. ‘Artificial’ Eggs in a Dish from Pluripotent Stem Cells

The aforementioned studies of human OSCs [73,97,98,99,101] also have immediate bearing on recent efforts to reconstitute the process of mammalian female gametogenesis, from primordial germ cells (PGCs) to fertilization-competent eggs, entirely ex vivo using pluripotent stem cells as starting material. This goal has recently been achieved with mouse embryonic stem cells (ESCs) and induced pluripotent stem cells (iPSCs) [117,118], albeit independent replication of the findings, which originally date back to 2012 [117], is still absent from the literature. Nevertheless, strides have been made in establishing a similar ability of human ESCs and iPSCs to generate PGC-like cells (PGC-LCs) in vitro [119,120], which can then be coaxed into immature oocyte-like cells that form follicle-like structures in vitro [101,121]. These observations, combined with advancements in the generation of human metaphase-II oocytes in vitro through improved follicle culture strategies [122], suggest that methods for the ex vivo production of human eggs are perhaps no longer in the realm of science fiction [123,124]. Notably, replacement of ESCs and iPSCs with OSCs in these types of studies may have several advantages. The first is that, unlike ESCs and iPSCs, OSCs are unipotent and wired from the start as a germ lineage, and therefore these cells require no directed differentiation or genetic manipulation to achieve a germline identity that is capable of oogenesis. Secondly, the specification of PGC-LCs from ESCs or iPSCs may fail to account for the importance of the germline mitochondrial DNA (mtDNA) bottleneck in ensuring maternal passage of ‘clean’ mtDNA generation after generation [125,126]. Indeed, reprogramming somatic cells that have accumulated years of mtDNA mutations and damage before conversion to iPSCs to reconstitute the female germ lineage for fertility reasons in women, if even feasible at some point in the future, could have disastrous consequences from a mtDNA inheritance perspective [127].

**Figure 3:**
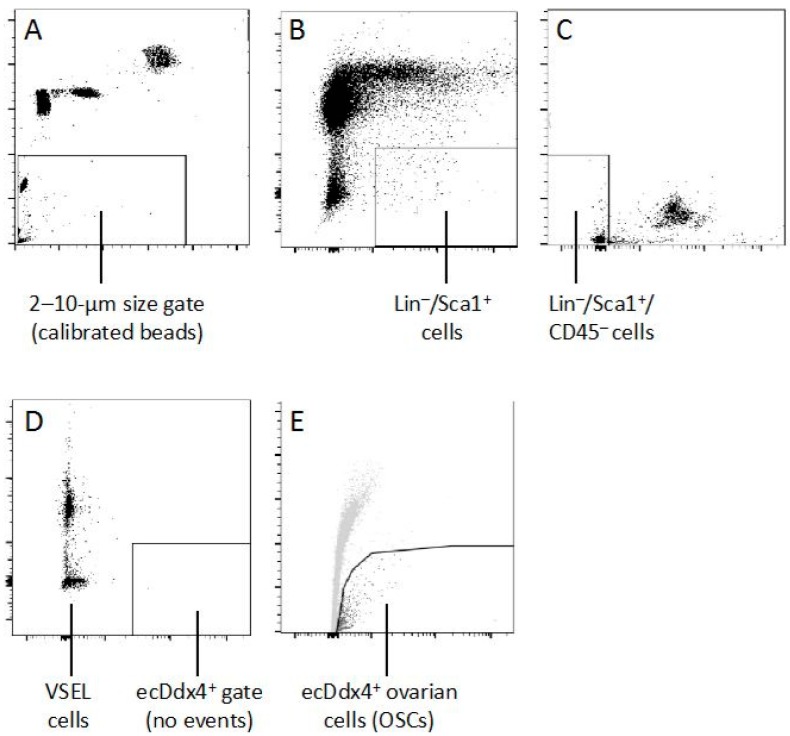
Mouse VSEL stem cells do not express externalized Ddx4. (**A**) To identify VSEL stem cells by FACS, size gates were initially set for 2–10 µm using calibrated beads. (**B**, **C**) Following dead cell exclusion, bone marrow-derived VSEL stem cells were identified in the 2–10 µm size gate as Lin^−^/Sca1^+^/CD45^−^ events, first by gating Lin^−^/Sca1^+^ cells followed by exclusion of CD45^+^ cells from this subpopulation (for additional details, see [129]). (**D**) Further analysis of the VSEL stem cell population shown in panel **C** using C-terminal-directed Ddx4 antibodies demonstrates that VSEL stem cells do not express externalized Ddx4 (ecDdx4^−^). (**E**) As a positive control for the VSEL stem cell analysis shown in panel **D**, parallel sorting of dispersed ovaries identifies viable ecDdx4^+^ cells, which represent OSCs (for additional details, see [68,73,112]). D.C. Woods and J.L. Tilly, unpublished data.

Before closing this discussion on the general topic of stem cells and oogenesis, it also is worth mentioning a commentary from Bhartiya et al. [128] regarding human OSCs, which voices the opinion that OSCs are more differentiated progenitor cells that initially arise from a pluripotent stem cell population referred to as very small embryonic-like (VSEL) stem cells originally identified in bone marrow by the Ratajczak laboratory over 10 years ago [129]. Although the existence of VSEL stem cells, like OSCs, has been questioned by some scientists [130,131], other groups have verified the presence of VSEL stem cells in adult tissues as well as the multi-potential nature of these cells in vivo and in vitro [132,133]. In 2008, Virant-Klun et al. [134] reported the presence of cells in the ovarian surface epithelium of post-menopausal women that possessed features of stem-like cells after in vitro cultivation. A population of cells with ESC-like properties was subsequently isolated from adult mouse ovaries [135], although these cells failed to exhibit the oogenic properties of isolated OSCs. A year later, VSEL stem cells were identified in adult mouse ovaries [136], eventually leading to a speculative proposal that VSEL cells are developmentally linked, and give rise, to OSCs [137]. It has been further proposed that both populations of cells express functional follicle-stimulating hormone (FSH) receptors [128,138], although OSCs were not actually isolated in the original study for direct comparative analysis of FSH receptor expression with VSEL stem cells. A similar claim that VSEL stem cells give rise to SSCs in the testis has also been put forth [139]. Unfortunately, direct experimental evidence establishing these hypothetical links of VSEL stem cells to either OSCs or SSCs is lacking, as is evidence from simple transplantation-based approaches used for functional characterization studies of SSCs [140,141,142] and OSCs [53,67,72,73,78,80,84,85] that VSEL stem cells can produce oocytes, embryos and offspring. Although future studies of VSEL stem cells may eventually provide such information, a key issue pertinent to the work of those labs presently studying human OSCs [73,97,98,99,101] is that the fundamental purification strategy used to isolate OSCs, which is based on cell surface exposure of the C-terminus of DDX4 [53,68,73,112], does not simultaneously isolate VSEL stem cells (Figure 3). Hence, the spectrum of downstream endpoints reported by many groups now working with purified OSCs reflect the characteristic features of specifically OSCs and not, as suggested by Bhartiya et al. [128], the properties of a mixed pool of OSCs and VSEL stem cells in culture.

## 5. Will Stem Cell-Derived Oocytes be of Future Value to Human Assisted Reproduction?

With multiple laboratories now confirming the existence and functional characteristics of OSCs in adult human ovaries [73,97,98,99,101], the most important question remains. Can these cells be utilized in some fashion to enhance, prolong or restore fertile potential in women? One possibility, which involves the use of patient-matched OSC mitochondria to invigorate eggs of women with a history of poor egg and embryo quality [102,103]—in a procedure termed autologous germline mitochondrial energy transfer (AUGMENT) [143,144], has recently been reviewed in detail elsewhere [104] and, thus, will not be addressed further here. A second obvious direction for this work, based on the guiding principles of regenerative medicine, involves development and testing of autologous transplantation-based approaches for prolongation or restoration of ovarian function. The in-vivo generation of new oocytes, capable of producing functional eggs, from transplanted OSCs has been conceptually validated by multiple independent groups using rodent models [53,67,72,73,78,80,84,85]. Moreover, preliminary findings from scientists at the National Institutes of Health indicate that comparable outcomes can be achieved with adult female non-human primates [145]. As important as these efforts are, we would like to focus our closing thoughts on what potentially could offer women faced with fertility challenges, due to insults or aging, unprecedented opportunities for having a genetically-matched child. This would arise from the development and use of OSC-based technologies designed to bioengineer reconstituted human ovarian tissue [101] for production of functional eggs from stem cells entirely ex vivo [105,106,107,108].

At a most basic level, three components would be needed for the development and testing of a platform to generate eggs from OSCs in vitro, and then evaluate the functional properties of eggs produced under these conditions. The first is a method to purify OSCs for introduction of a traceable reporter (e.g., green fluorescent protein, GFP); the second is a method to deliver GFP-expressing cells back into an environment that enables their differentiation into oocytes, which then form primordial follicles; the third is a method to grow primordial follicles to small antral stages so that the enclosed cumulus-oocyte complexes can be collected for in-vitro maturation (IVM) of the oocytes to produce metaphase-II eggs. Using adult human ovarian cortical tissue, we have recently established feasibility of the first two components [73], both of which have been independently verified [97]. However, the ability of OSC-derived human oocytes, enclosed within primordial and transitional-primary follicles, to complete maturation to the metaphase-II stage has not yet been shown. Nonetheless, past studies using bovine, non-human primate and human ovarian cortical tissues have reported successful in vitro growth of ‘endogenous’ primordial or primary (unilaminar) follicles to early antral stages, yielding oocytes for IVM [122,146,147,148,149]. Moreover, numerous clinical studies now exist that describe methods for IVM of human germinal vesicle-stage immature oocytes to mature eggs, which then fertilize to produce viable embryos for transfer [150,151,152,153,154,155,156,157]. If these published technologies can be effectively interwoven into a single sequential platform, such an approach should, at least theoretically, enable the generation of eggs from OSCs introduced into ovarian cortical strips or re-aggregated with dispersed ovarian tissue as a suitable microenvironment for growth and differentiation [107,108].

However, two key issues must be taken into consideration here. The first is the source of the ovarian cortical tissue to be used to ‘house’ the purified OSCs for differentiation. Clinically, the use of autologous tissue (viz. patient matched OSCs and ovarian tissue) would be optimal, although ovaries may become unsupportive of OSC differentiation and follicle formation with advancing age [84]. One potential way around this problem would be to specify undifferentiated or pre-granulosa cells from patient-matched iPSCs, analogous to what has been reported using mouse ESC models [158,159] and recently extended to human iPSCs [159], that could be aggregated with OSCs and other ovarian somatic cells to reconstitute autologous ovarian tissue ex vivo. Efforts to parallel these types of experiments with an animal model that does not have limited ovarian tissue availability, such as the bovine model in which OSCs have recently been identified ([93,94]; Woods DC and Tilly JL, unpublished) and primordial follicles have been grown to antral stages ex vivo [147], will probably prove invaluable for troubleshooting each step as the multiple strategies are brought together into a unified platform. Use of cow ovaries may also help address the second key issue, which is related to testing the competency of eggs generated from stem cells entirely outside of the body. While human eggs produced from OSCs in vitro, if established as feasible, can be comparatively evaluated with ‘endogenous’ eggs for various endpoints, such as metabolic profiles, –omics and parthenogenetic potential, bovine eggs produced by stem cell-based technologies could be assessed completely through fertilization, embryonic development and live-birth offspring. Although there is a long road ahead to realizing this type of technological advancement, which may seem like fantasy at present, we should not lose track of the fact that both postnatal oogenesis and reconstitution of the entire life cycle of the female germline in vitro in mammals were viewed as science fiction less than a decade or two ago. Time will tell if current science evolves into tomorrow’s practice [107], but the recent work with OSCs from multiple laboratories is exciting and, we feel, at the forefront of scientific discovery efforts in the field of human reproductive biology and assisted reproductive technologies.
